# The Impact of Parent–Child Attachment on School Adjustment in Left-behind Children Due to Transnational Parenting: The Mediating Role of Peer Relationships

**DOI:** 10.3390/ijerph19126989

**Published:** 2022-06-07

**Authors:** Huilan Zhang, Chunkao Deng

**Affiliations:** School of Education, Wenzhou University, Wenzhou 325035, China; 21460410022@stu.wzu.edu.cn

**Keywords:** school adjustment, parent–child attachment, left-behind children, peer relationships

## Abstract

In China’s eastern coastal areas, the transnational parenting of left-behind children creates a distinct form of left-behind child. Previous research has indicated that children who have been left behind have a low degree of school adjustment. The major purpose of this research was to investigate the impact of parent–child attachment on school adjustment in children left behind by migrant parents, as well as the mediating role of peer relationships in this process. The parent–child attachment section of the Inventory of Parent and Peer Attachment (IPPA), the Adaptation subscale of the Adolescent Mental Health Quality Questionnaire—Chinese Version (AMHQQ-C), and the Student Peer Relationship Scale (SPRC) were used to survey 405 left-behind children in grades 3–6 of seven elementary schools in the hometowns of overseas Chinese parents from Zhejiang Province. It was discovered that, compared to non-left-behind children, left-behind children showed lower levels of parent–child attachment and school adjustment, while peer relationships appeared polarized. In addition, parent–child attachment and peer relationships considerably predicted the level of school adjustment in children left behind due to transnational parenting. More importantly, the mediation analysis revealed a partial mediating effect of peer relationships on the linkages between parent–child attachment and school adjustment among children who were left behind in transnational foster care.

## 1. Introduction

School adjustment is a dynamic process in which students seek to adapt to school demands, as well as a state in which they happily participate in school activities and achieve academic success [1]. In studies on children’s school adjustment, researchers generally agree that academic, behavioral, and emotional adjustment are all part of school adjustment. Academic adjustment refers to how students cope with homework, classwork, and tests; behavioral adjustment refers to how students behave at school; and affective adjustment refers to how students feel and act at school [2]. Good school adjustment helps children to complete school successfully, form positive and friendly peer relationships, and develop social competence [3]. However, assessment of the current school adjustment status of Chinese children is not optimistic, with many students suffering from mild-to-severe school maladjustment [4]. As a result, it is critical to understand the factors that influence children’s school adjustment, as well as the underlying mechanisms of action.

With the reform and opening up of China, a large number of rural residents have flocked to first- and second-tier cities, leaving their children behind in their hometowns under the supervision of one parent or grandparents; these children are known as “left-behind children.” School adjustment in left-behind children is worse than average because of their unique family backgrounds. Wang. F. used children’s self-assessment to examine the school adjustment status of 304 rural left-behind children in six cities in Hubei Province, and the results were unsatisfactory [5]. Sun, X.J. et al., on the other hand, used teacher ratings to compare school adjustment in left-behind children and non-left-behind children [6]. They found that the overall level of school adjustment in left-behind children was much lower; moreover, among students with good school adjustment, the number of left-behind children was lower than that of non-left-behind children. The school adjustment status of left-behind children experiencing transnational parenting may have its own characteristics, defining a distinct type of left-behind child in China’s eastern coastal region.

Children under the age of 18 who have both parents (new overseas Chinese) and live with family members in their domestic overseas Chinese hometowns are referred to as transnational foster left-behind children. Although parents going abroad to work can increase their economic income and create a material foundation for the education of left-behind children, due to family separation and a lack of parental care, researchers believe that left-behind children are generally poorly adapted to school, and are more likely to have academic and behavioral problems [7]; moreover, it is believed that their school adaptation is closely related to their psychological state [8]. In terms of academic adjustment, Chen, X. et al. revealed that about 75% of the children who were left behind by migrant parents had poor academic performance [9]. Ezaki discovered that left-behind children in Nepal performed poorly at the elementary school level [10]. In terms of behavioral adaptation, researchers discovered that transnational left-behind children were more likely to engage in harmful behaviors such as smoking, playing video games, and skipping classes, while problematic behaviors such as truancy were more common among overseas transnational left-behind children [11]. In terms of emotional adaptation, comparing transnational left-behind children to inter-provincial left-behind children and intra-provincial left-behind children, Leskauskas found that children left behind due to their parents travelling abroad had the poorest mental health status and the worst psychological status [12]. Previous studies have discussed the current situation regarding school adaptation in left-behind children in overseas Chinese hometowns, but the impact mechanism caused by their special status is not clear.

Based on ecosystem theory [13], individual development is nested within a series of interacting environmental systems that influence development. The family, as the micro-environment that most directly affects children, is the first environmental system that influences the psychological development of individuals. Compared to other family variables, the parent–child attachment is a more comprehensive indicator of a child’s internal emotional connection to their parent and their external attachment behaviors [14]. Parent–child attachment is a reciprocal, enduring physical and emotional relationship that exists throughout childhood and into adulthood [15]. Belsky, J. suggested that parent–child attachment can be an important predictor of children’s future behavioral adjustment [16]. It has been shown that parent–child attachment is associated with the level of school adjustment in mobile children. Bardack, S. discovered a significant positive relationship between school adjustment and parent–child attachment levels among mobile adolescents, with higher maternal attachment scores associated with better school adjustment; similar conclusions were drawn for paternal attachment [17]. In a study by Jin, Z., it was discovered that the level of parent–child attachment directly influenced the urban adjustment status of migrant children, and that mental toughness as a mediating variable indirectly influenced the urban adjustment status of migrant children [18]. Parent–child attachment is thought to have a predictive effect on mobile individuals’ school adjustment. In comparison to ordinary children and rural migrant children, children left behind due to their parents travelling abroad have a unique mobility trajectory, namely: leaving the country—returning to the country—leaving the country—returning to the country. In conclusion, left-behind children in the diaspora are also a type of mobile child, so the important factors that influence their school adjustment may be similar. As a result, we proposed Hypothesis 1.

**Hypothesis** **1** **(H1):** *Parent–child attachment is substantially connected with school adjustment.*

Based on the developmental theory of group socialization [19], children’s socialization is the process of their acceptance by the society in which they live, and their shared environment with peers can have a more direct and far-reaching impact on their psychological development than their families. A peer relationship is a collaborative interpersonal relationship that arises and develops in the course of interaction among children of similar ages, or of comparable levels of psychological development. The two main components of peer relationships are peer acceptance, which is a group-oriented one-way structure reflecting the group’s attitude toward the individual, and friendship, which is an individual-oriented two-way structure reflecting the emotional connection between individuals [20]. Peer interactions have been demonstrated, in numerous studies, to be major predictors of children’s school adjustment. The number of peers is highly related to the level of school adjustment, and an adolescent’s status in peer groups predicts their academic achievement [21]. Ryan, A.M. found that when children initially enter school, their peer interactions predict their school achievement one year later [22]. Compared to friendship, peer acceptance was a stronger predictor of academic adjustment. Children who are accepted and liked by their peers perform better academically, whereas children who are rejected by their peers perform worse academically and have higher rates of dropping out. Because of the incomplete family structure of left-behind children with one or both parents absent, peer groups become their main object of interaction. Left-behind children in transnational adoptions have higher emotional demands than the ordinary child, and the majority of left-behind children want to interact with their peers. Based on ecosystem theory, peer relationships, as an external environmental factor, may influence school adjustment in left-behind children by affecting the family as the first environmental system. Therefore, we proposed Hypothesis 2.

**Hypothesis** **2** **(H2):** *Peer relationships play a mediating role between parent–child attachment and school adjustment.*

Studies have explored the current school adjustment situation of left-behind children experiencing transnational parenting. Furthermore, they have confirmed the effects of parent–child attachment and peer relationships on school adjustment in children in general and in migrant children; however, few studies have addressed the mechanisms of school adjustment in left-behind children experiencing transnational parenting. Accordingly, this research aims to explore the mechanisms and effects of parent–child attachment on school adjustment in transnational left-behind children. The findings can be used to help left-behind children in the future.

## 2. Methods

### 2.1. Participants

A whole-group sampling method was used to select students in grades 3–6 from three schools in Wenzhou, one of the cities with a large number of transnational left-behind children in China’ s eastern coastal areas. In order to reduce the negative perception of children left behind due to transnational parenting, all students were asked to fill in the questionnaire instead of only the children left behind. A total of 612 children participated in the questionnaire survey; of these children, 18 did not complete the questionnaire; thus, altogether, 594 participants took part in the survey. Among them, 405 left-behind children (53.3% female; mean = 10.32 years, SD = 0.91, range 9–12 years) were identified through their answers to “your parents’ absence”. Data from these left-behind children were used for the final analysis in this study. Among the places of birth, students born in their own hometowns accounted for the largest proportion, at 46.67%. Those born abroad accounted for 40.74%, and others accounted for 12.59%. Approximately 37.88% of the children had been left behind for 1–2 years; 35.64% of the children had been left behind for 3–5 years; and 26.48% of the children had been left behind for over 5 years.

### 2.2. Procedure

Prior to testing, consent was obtained from the principal and teachers for this study. All questionnaires were administered using group tests, with trained graduate students as the main testers in each class, and with the assistance of the classroom teacher. If the participants had any questions, the researchers would patiently provide explanations to them. After the questionnaires were returned, common method bias tests, descriptive statistics, and correlation analyses were conducted using SPSS 26.0, and a mediation effects analysis was conducted using PROCESS for SPSS, developed by Hayes (2013) [23].

### 2.3. Measurements

#### 2.3.1. Parent–Child Attachment Questionnaire

The Chinese version (Zhao, J., 2019) [24] of the parent–child attachment questionnaire (Armsden and Greenberg, 1987) [25] was used to evaluate the attachment between the students and their parents. The questionnaire consists of 20 items, divided into three dimensions—trust, communication, and detachment—and scored on a 5-point scale, with 1 indicating “never” and 5 indicating “always”. The total parent–child attachment score is the sum of the scores of the trust and communication dimensions minus the detachment dimension score. In this study, the internal consistency coefficient of the parent–child attachment questionnaire was 0.938, and the validation factor analysis showed that χ^2^/df = 3.752, CFI = 0.989, TLI = 0.971, and RMSEA = 0.065.

#### 2.3.2. School Adaptation Scale

The adaptation subscale of the Adolescent Psychological Inventory (Zhang, D.J., 2012) [26] was used to evaluate students’ school adaptation. The questionnaire consists of 13 items and is scored on a 5-point scale, with 1 indicating “not at all” and 5 indicating “completely”. In this study, the internal consistency coefficient of the school adjustment scale was 0.892, and the validation factor analysis showed that χ^2^/df = 4.933, CFI = 0.930, TLI = 0.924, and RMSEA = 0.042.

#### 2.3.3. Student Peer Relationship Scale

The Student Peer Relationship Scale (Asher, 1986) [27] was used to evaluate the relationships between students and their peers. The questionnaire consists of 16 items and is scored on a 5-point scale, with 1 indicating “not at all” and 5 indicating “completely”. The scale is divided into three dimensions: welcome, exclusion, and loneliness. After converting the reverse scores of the 16 items, students’ total scores on the 16 items were calculated, and the higher the score, the better the peer relationship. In this study, the internal consistency coefficient of the Student Peer Relationship Scale was 0.930, and the validating factor analysis showed that χ^2^/df = 2.473, CFI = 0.944, TLI = 0.901, and RMSEA = 0.081.

## 3. Results

### 3.1. Common Method Bias Test

In this study, the common method deviation was controlled by adopting anonymous measurement and partial item reverse measures. Nevertheless, common method bias might exist in the results collected from the self-reported questionnaires. Therefore, the Harman one-way test was used to test for common method bias. The results showed that there were 13 factors with eigenvalues greater than 1, and the variance explained by the first factor was 28.677%, which was less than the critical criterion of 40%; this indicates that the possibility of common method bias in this study was low.

### 3.2. Descriptive Statistics and Correlation Analysis of Latent Variables

The results of the mean and standard deviation descriptive statistics for each study variable are shown in Table 1. For parent–child attachment, there were significant differences in the dimensions of communication (*t* = −2.62, Cohen’s d = −0.70, *p* < 0.01) and trust (*t* = −0.35, Cohen’s d = −0.35, *p* < 0.05) between children left behind by migrant parents and children not left behind, and the former scored lower. In addition, left-behind children showed a stronger level of detachment (*t* = −1.56, Cohen’s d = 0.58, *p* < 0.01). The differences in school adjustment were also significant (*t* = −5.64, Cohen’s d = −0.73, *p* < 0.001); in terms of peer relationships, compared to non-left-behind children, the left-behind children scored lower in welcoming (*t* = −3.19, Cohen’s d = −0.47, *p* < 0.01) and higher in loneliness (*t* = −2.59, Cohen’s d = 0.38, *p* < 0.01).

To better control for errors, the correlation coefficients between variables were estimated using a correlation analysis of latent variables. The latent variables used were parent–child attachment, school adjustment, and peer relationships. The results showed that the model fit index was good, with χ^2^/df = 4.84, CFI = 0.92, TLI = 0.90, and RMSEA = 0.07. The results of the correlation analysis showed that parent–child attachment, school adjustment, and peer relationship were significantly correlated with each other (*p* < 0.01). Specific results are shown in Table 2.

### 3.3. Testing the Direct Predictive Effect of Parent–Child Attachment on School Adjustment

Parent–child attachment, school adjustment, and peer relationships were significantly correlated with each other, so a structural equation model was constructed using the PROCESS plug-in; it controlled for variables such as the gender and grade level of the study participants, with school adjustment as the dependent variable and parent–child attachment as the independent variable, to test the direct effect of parent–child attachment on school adjustment. The results showed that the model fit index was good (χ^2^/df = 2.284, CFI = 0.940, TLI = 0.955, and RMSEA = 0.068). Parent–child attachment significantly and positively predicted students’ school adjustment (β = 0.476, *p* < 0.001).

### 3.4. Testing the Mediating Role of Peer Relationships

**Figure 1:**
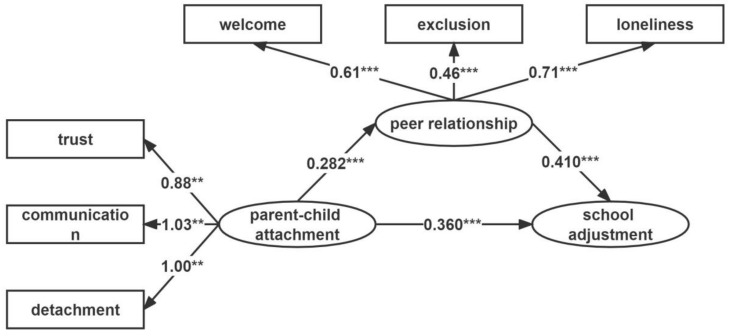
The model of peer relations mediating the relationship between parent–child attachment and school adjustment. ** *p* < 0.01, *** *p* < 0.001.

To further examine the mechanism of the role of parent–child attachment in students’ school adjustment, peer relationships were included as mediating variables in the model for testing (Figure 1). The results showed that the model fitted well, with good indices (χ^2^/df = 2.806, CFI = 0.894, TLI = 0.911, and RMSEA = 0.054). Parent–child attachment significantly and positively predicted peer relationships (β = 0.282, *p* < 0.001) and peer relationships significantly and positively predicted school adjustment (β = 0.410, *p* < 0.001).

A mediating effect analysis was conducted using a deviation-corrected percentile bootstrap test. The results showed that the 95% confidence interval of the mediating effect of peer relationship between parent–child attachment and school adjustment was [0.073, 0.166], not including 0, and the mediating effect was significant, with a mediating effect value of 0.24 (see Table 3). This indicated that peer relationships partially mediated the relationship between parent–child attachment and school adjustment.

## 4. Discussion

### 4.1. Left-behind Children Have Lower Levels of School Adjustment and Parent–Child Attachment

The analysis of the differences among school adjustment, peer relationship, and parent–child attachment between LBCs in transnational foster care and NLBCs found that, first, in terms of school adjustment, children left behind in transnational foster care scored lower than children left behind in non-families; this was especially true in the academic adaptation dimension, which is consistent with previous related studies [28]. This may be due to the long-term absence of parents negatively affecting the individual’s school adjustment. Second, the significant polarization of the peer relationships of left-behind children was related to the two-sided peer relationship. Last, in terms of parent–child attachment, children left behind in transnational foster care scored higher in the negative dimension and lower in the positive dimension than children not left behind. Previous studies have found that parental absence had a negative predictive effect on children’s loneliness [29]. This may be the result of weaker parent–child communication after transnational separation.

Compared with the non-left-behind children, the left-behind children experienced parent–child separation in childhood, which lead to their low level of parent–child attachment. A child’s personality development is based on a healthy home environment, whereas the contrary is more likely to result in personality flaws and deviant behaviors. Children who have experienced parent–child separation, emotional neglect, educational neglect, and other adverse family environments are prone to developing bad behavioral habits and extreme personalities [30]. Therefore, they have lower levels of school adaptation and parent–child attachment.

### 4.2. Parent–Child Attachment Directly Affects School Adjustment in Left-behind Children in Transnational Parenting

This study explored the relationship between parent–child attachment and school adjustment in left-behind children experiencing transnational parenting. The results of the latent variable correlation analysis showed that parent–child attachment was significantly and positively related to the school adjustment level of left-behind children in transnational foster care. Meanwhile, after controlling for gender and grade level, structural equation modeling indicated that parent–child attachment had a positive predictive effect on school adjustment in transnational left-behind children. These findings confirmed research Hypothesis 1, which stated that parent–child attachment would positively affect school adjustment in left-behind children. Ratelle, C.F. et al. also found that positive mother–child attachment could significantly predict children’s academic and emotional adjustment, and father–child attachment also had the same effect [31]. Notably, the direct predictive effect of parent–child attachment remained significant after the introduction of peer relationships as a mediating variable. This suggests that the family, as a micro-environmental system for child development, not only has a direct effect on school adjustment in children left behind in transnational foster care, but also acts partly as a “bridge” through the peer relationship variable; this is consistent with the ecosystem theory.

Attachment between parents and children is the first aspect that effects a child’s development. Mother–child attachment is more important. Chen, M. et al. found that mother immigrants were more likely to lead to maladjustment in left-behind children [32]. In this study, from the perspective of parent–child communication, parents abroad lacked sufficient trust in their children left behind in transnational foster care, and their communication attitude was generally domineering. In addition, their communication was primarily about school and life, and the medium of communication was primarily online. Thus, children lost the motivation to communicate, and they scored lower than non-left-behind children on the communication dimension (d = −0.70).

### 4.3. The Mediating Role of Peer Relationships in Parent–Child Attachment and School Adjustment

Peer relationships were found to mediate the relationship between parent–child attachment and school adjustment in children left behind in transnational foster care. The higher the level of parent–child attachment in children left behind in transnational foster care, the higher their level of school adjustment when peer relationships were positive. This finding supports both research Hypothesis 2 and the ecosystem theory. Family and peers play a role in children’s growth and development as two independent “social systems”, and their influences on children’s behavior are also independent of each other [33].

Firstly, the level of parent–child attachment has an impact on the peer relationships of children who have been left by their parents because of transnational parenting. Internal working-model representations of early parental attachment in children serve as prototypes for the development of other social relationships later in life [34]. Although parent–child attachment may no longer be dominant in children’s social development, good parent–child relationships mitigate the effects of poor peer relationships. Children also feel trust in others after actively resolving conflicts of trust and mistrust, according to Erikson’s eight stages of psychosocial development. The low level of parent–child attachment among children left behind in transnational parenting has a negative effect on the development of positive peer relationships during adolescence.

Additionally, peer relationships influence school adjustment in left-behind children in transnational parenting. Peers are an important source of support when teens face environmental challenges; at school, this refers to classmates. They support each other in learning, share with each other in life, and actively communicate with each other emotionally. Additionally, good peer relationships may even compensate for children’s unmet attachment needs in parent–child relationships [35]. Children left behind in transnational adoptions feel a sense of belonging to the group, which, in turn, affects their academic, behavioral, and emotional adaptation status.

Lastly, through peer relationships, the level of parent–child attachment influences school adjustment in children left behind in transnational foster care. Parent–child interactions in transnationally raised, left-behind families are mostly replaced by interactions with surrogate guardians, which cannot fully replace the true parent–child interaction function, according to the spillover hypothesis. As a result, the partial spillover of transnationally raised, left-behind children’s family systems into the school system increases their peer group interactions, and peer relationships become increasingly important.

It is worth noting that there are two sides to the peer relationships of children left behind in transnational parenting. Peer relationships have a predictive effect on students’ school adaptation [36]. At the same time, peer relationships will play a compensatory role in parent–child attachment, and good peer relationships can improve the parent–child insecure attachment style formed in the early stage of the individual [37]. However, bad peer relationships can be detrimental to a child’s development. Many studies have confirmed that deviant peer affiliation can increase individual problem behaviors such as aggression [38]. Each peer group has its own subculture and set of values, which may include a negative group culture that is diametrically opposed to the school–family relationship. Children who have been abandoned by their parents and lack parental guidance are more likely to pursue extreme peer acceptance and develop negative behaviors.

## 5. Conclusions

This research examined the relationship between parent–child attachment and school adjustment in Chinese transnational left-behind children. Parent–child attachment in the family environment and peer relationships in the school environment were important factors influencing school adjustment. Moreover, peer relationships contributed to the moderating relationship of school adjustment. The findings indicated that parent–child attachment was a critical component affecting transnational left-behind children’s school adjustment, and that peer relationships acted as a moderator in the path between parent–child attachment and school adjustment.

## 6. Limitations and Future Research Directions

There are shortcomings in this study. First, the study sample mainly comprised children in grades 3–6; future studies can expand the scope to further explore the effects on left-behind children at different ages. In addition, this study used a cross-sectional research design; future studies can further explore a longitudinal design and cross-lagged analysis.

## Figures and Tables

**Table 1:** Descriptive statistics of each variable of transnational parenting of left-behind children (LBCs) and non-left-behind children (NLBCs) (n = 594).

	LBCs (M ± SD)	NLBCs (M ± SD)	Cohen’s d	*t*
Communication	3.30 ± 0.45	3.61 ± 0.44	−0.70	−2.62 **
Trust	3.59 ± 0.42	3.77 ± 0.59	−0.35	−2.01 *
Detachment	3.81 ± 0.39	3.59 ± 0.37	0.58	−1.56 **
Welcome	3.44 ± 0.47	3.69 ± 0.57	−0.47	−3.19 **
Loneliness	4.06 ± 0.58	3.87 ± 0.40	0.38	−2.59 **
Exclusion	4.18 ± 0.89	4.08 ± 0.91	0.11	−1.40
Parent–child attachment	3.28 ± 0.46	3.66 ± 0.42	−0.86	−2.50 ***
Student Peer Relationship	3.80 ± 0.53	3.98 ± 0.62	−0.31	−2.71 **
School adjustment	3.29 ± 0.45	3.70 ± 0.47	−0.73	−5.64 ***

The total number of individuals (population) = N; arithmetic mean = M; standard deviation = SD. * *p* < 0.05, ** *p* < 0.01, *** *p* < 0.001.

**Table 2:** Analysis of correlations among variables in transnational parenting of left-behind children (n = 405).

	1	2	3	4	5	6	7	8	9
1. Parent–child attachment	1								
2. Peer relationships	0.33 **	1							
3. School adjustment	0.54 **	0.54 **	1						
4. Communication	0.95 **	0.32 **	0.51 **	1					
5. Trust	0.91 **	0.29 *	0.55 ***	0.79 **	1				
6. Detachment	0.67 **	0.26 **	0.26 **	0.53 **	0.44 **	1			
7. Welcome	0.32 **	0.86 **	0.57 *	0.32 **	0.29 **	0.19 **	1		
8. Loneliness	0.30 **	0.93 **	0.47 *	0.29 **	0.26	0.22 **	0.69 **	1	
9. Exclusion	0.27 **	0.89 **	0.41 ***	0.25 ***	0.24 *	0.23 **	0.62 **	0.80 *	1

* *p* < 0.05, ** *p* < 0.01, *** *p* < 0.001.

**Table 3:** Significance test of mediating effect and mediating effect value.

	Effect Value	Standard Error of Indirect Effect	95% Confidence Interval		Amount of Effect (%)
			Lower Bound	Upper Limit	
Total effect	0.476 ***	0.042	0.395	0.556	-
Indirect effect	0.116 ***	0.023	0.073	0.166	24.37
Direct effect	0.360 ***	0.040	0.282	0.438	75.63

*** *p* < 0.001.

## Data Availability

The datasets generated and analyzed during the current study are available from the corresponding author upon reasonable request.
